# Probable mechanisms of biocidal action of *Cocos nucifera* Husk extract and fractions on bacteria isolates

**DOI:** 10.1186/s12906-015-0634-3

**Published:** 2015-04-14

**Authors:** David A Akinpelu, Kazeem A Alayande, Olayinka A Aiyegoro, Oluseun F Akinpelu, Anthony I Okoh

**Affiliations:** Department of Microbiology, Obafemi Awolowo University, Ile Ife, Osun State Nigeria; GI Microbiology and Biotechnology Unit, Agricultural Research Council, Animal Production Institute, Irene 0062, Pretoria, South Africa; Department of Biological Science, Faculty of Agriculture and Technology, North West University, Mafikeng Campus, South Africa; Applied and Environmental Microbiology Research Group (AEMREG), Department of Microbiology and Biochemistry, University of Fort Hare, Alice, 5700 South Africa; SA-MRC Microbial Water Quality Monitoring Centre, University of Fort Hare, Alice, 5700 South Africa

**Keywords:** *Cocos nucifera*, Fractions, Minimum inhibitory concentrations, Rate of killing, Protein leakage potassium ion leakage, Nucleotide leakage

## Abstract

**Background:**

The incidence of resistance to the existing antibiotics by microorganisms demand increased effort in the development of new antibiotics for the treatment of microbial infections and diseases. Infections due to multidrug resistant pathogens are difficult to manage due to relatively limited choices of antimicrobial agents. This study investigated antimicrobial activities of the husk extract of *Cocos nucifera* on some bacteria that are associated with human diseases.

**Methods:**

Powdered husk of *Cocos nucifera* was cold extracted using mixture of methanol and distilled water in ration 3:2 (v/v). Extract was partitioned into n-hexane. Chloroform, ethylacetate and n-butanol fractions and thereafter, the minimum inhibitory concentrations (MICs) of the extract and those of the fractions were determined. The ethylacetate fraction was found to be more active and was partially purified by a combination of thin-layer and column chromatography. Finally, the rate of killing, leakages of proteins, potassium ions and nucleotides from the tests bacterial cells were determined.

**Results:**

The minimum Inhibitory concentrations (MICs) of the extract ranged between 0.39 and 12.50 mg/ml and those of the fractions ranged between 0.16 and 5.00 mg/ml. The time-kill assay revealed a minimum of 27.8% killed at 1 × MIC after 15 min contact time with the fractions and a minimum of 95% killed after 120 min.

Varying amount of proteins, potassium ions as well as nucleotides were leaked from selected bacterial isolates by the four active fractions. The amount of proteins leaked from the cells after 15 min contact time ranged between 3.56 and 19.08 μg/ml at 1 × MIC and between 10.97 and 19.54 μg/ml at 2 × MIC. The amount of potassium ions leaked from the cells after 15 min contact time ranged between 0.182 and 0.379 mg/ml at 1 × MIC and between 0.227 and 0.561 mg/ml at 2 × MIC. The nucleotides leaked from the cells after 15 min contact time ranged between 0.609 and 2.446 μg/ml at 1 × MIC and between 0.897 and 2.841 μg/ml at 2 × MIC.

**Conclusions:**

This study established the possibility of developing antimicrobial agents of natural origin to combat resistance to antimicrobial compounds by some pathogens currently being experienced in agricultural and health care environments.

## Background

The bacterial cytoplasmic membrane is a very subtle organelle and is extremely active metabolically. It acts essentially as a selective permeability barrier between the cytosol and cell’s peripheral environment [1]. Some groups of antimicrobial compounds disrupts bacterial cell membrane activity; as either potassium ion leakage and/or leakage of material that absorbs at 260_nm_ from within the cell [2]. Any membrane active therapeutic agent can cause damage by action upon either the membrane potentials, bound enzymes or permeability.

As resistance to antibiotics becomes more common; the prevalence of multidrug-resistant (MDR) pathogens in clinical and veterinary practices thus increases [3], therefore, new and even more powerful agents are needed for the treatment of MDR microbial infections. This has now led to an intensive search for newer and more effective antimicrobial agents to deal with these problems. Such agents are now been sourced from bioactive components of medicinal plants.

Medicinal plants are of great importance to the health of individuals and communities because of innate phytochemicals that initiates a definite physiological action on the human body. Plants can synthesis many different types of secondary metabolites, which have been subsequently exploited by humans to their advantages in a diverse array of applications [4]. One of the great advantages of medicinal plants is the availability and often very low side effects after ingestion [5].

Our candidate plant for this study: *Cocos nucifera*, is a member of the family Arecaceae, the only accepted species in the genus *Cocos*. It is a large palm, growing up to 30 meters tall, with pinnate leaves 4–6 meters long, and pinnae 60–90 cm long.

Traditionally the juice of the young spandex when fresh is used in the managements of diarrhoea and diabetes [6] and it is also excellently active against different pathogenic bacteria causing several life-threatening infections to humans [7]. The polyphenolic compound extracted from husk fiber of *Cocos nucifera* exhibited antibacterial and antiviral activities against some isolates and it also inhibited lymphocyte proliferation [7]. The methanolic extract of the husk of *Cocos nucifera* has also been reported to have produced *in vitro* antimicrobial activity against all strains of *Staphylococcus aureus* tested [8]. Extract from this plant also showed antifungal activity against *Microsporum canis, M. gypseum, M. audouinii, Trichophyton mentagrophytes, T. rubrum, T. tonsurans* and *T. violaceum* [9]. It also exhibited anti-nociceptive and anti-inflammatory activities which confirm the popular use of this plant in several inflammatory disorders as reported by Rinaldi and co-workers [10].

Both the literature and ethno-botanical records indicate a general consensus on the use of potent antimicrobial medicinal plants to provide cheaper drugs that may complement existing supplies from orthodox medicine. Traditionally herbal or alternative medicine is extensively practiced in the prevention and treatment of various infectious illnesses and it has regained public attention in recent years most especially in the developing countries because it is relatively cheaper and easily accessible. This study is therefore designed to investigate the probable mechanisms of action of different potent fractions of the extract on *K. pneumoniae* representing the Gram negative bacteria and *E. faecalis* representing Gram positive bacteria.

## Methods

### Microorganisms

Microorganisms used in this study were obtained from culture collections of Microbiology Department, Obafemi Awolowo University, Ile-Ife, Osun State, Nigeria. These organisms include typed cultures of National Collection of Industrial Bacteria (NCIB) and locally isolated organisms (LIO). The bacterial isolates used include:

### Gram positive

*Bacillus cereus* (NCIB 6349), *Bacillus polymyxa* (LIO), *Bacillus anthracis* (LIO), *Bacillus subtilis* (3610), *Bacillus stearothermophillus* (NCIB 8222), *Clostridium sporogenes* (NCIB 532), *Corynebactarium pyogenes* (LIO), *Staphylococcus aureus* (NCIB 8588), *Enterococcus faecalis* (NCIB 775) and *Micrococcus luteus* (NCIB 196).

### Gram negative

*Escherichia coli* (NCIB 86), *Klebsiella pneumoniae* (NCIB 418), *Pseudomonas aeruginosa* (NCIB 950) and *Pseudomonas flourescens* (NCIB 3756).

### Culture media used

Nutrient broth and nutrient agar (LAB M® Lancashire BL97JJ, UK) were used for subculturing the organisms while Mueller-Hinton agar (LAB M® Lancashire BL97JJ, UK) was used for sensitivity testing. The media were sterilized using autoclave at 121°C and 1.05 kg/cm^3^ for 15 minutes.

### Drying and extraction of the plant sample

The husks of *Cocos nucifera* used in this study were collected around Ile Ife, Osun State, Nigeria. The plant sample was identified by Professor HC Illoh of the Department of Botany, Obafemi Awolowo University, Ile-Ife, Nigeria. Voucher specimen of the plant sample with voucher number IFE 1742 was prepared and deposited at the herbarium of the Botany Department for references. The husk was dried in hot-air oven at 45°C until a constant weight of the sample was reached. This was ground into fine powder. Exactly 1.5 kg of the powdered sample was extracted with methanol/sterile distilled water in ratio 3:2 (v/v) for four days with regular agitation. The supernatant collected was filtered into a clean sterile dried conical flask. The filtrate was concentrated *in vacuo* and lyophilized. The yield obtained was 110.5 g, dark brown in colour.

### Solvent partitioning of the husk extract of *Cocos nucifera*

Exactly 100 g of the husk extract was resolved into 200 ml of sterile distilled water and poured in a separatory funnel and extracted in succession with organic solvents according to order of polarities, starting with the least polar solvent. The resulting n-hexane fraction was concentrated to dryness *in vacuo* and the residue (22.50 g) was kept in a freezer in an air-tight container. The resultant aqueous phase was re-concentrated *in vacuo* to remove traces of n-hexane. The residue was further extracted with chloroform (4 × 200 ml). The chloroform fraction obtained was also concentrated *in vacuo* to dryness and 3.5 g powder collected was kept in freezer for further use. The ethylacetate (25.15 g) and n-butanol (17.5 g) fractions were also obtained using similar procedure. The remaining aqueous fraction was freeze-dried to yield 32.65 g powder which was kept in the freezer for further use.

### Determination of minimum inhibitory concentrations (MICs) of the husk extract of *C. nucifera* and the fractions on bacterial isolates

The MICs of the extract and the fractions obtained was determined using the method described by Akinpelu and Kolawole [11]. Two-fold dilution of the extract and fractions were prepared separately and 2 ml of different concentrations of the solution was added to 18 ml of pre-sterilized molten nutrient agar to give final concentrations regimes of 0.391 to 25.0 mg/ml. The medium was then poured into sterile Petri dishes and allowed to set. The surfaces of the media were allowed to dry before streaking with 18 h old standardized bacterial cultures. The plates were later incubated at 37°C for up to 72 h after which they were examined for the presence or absence of growth. The MIC was taken as the lowest concentration that prevented the growth of the bacteria.

### Determination of rate of kill on the test isolates by the active fractions

The rate of kill was determined using the method described by Odenholt [12] with some modification. Experiment was performed using each of the active fractions on the viability of *Enterococcus faecalis* representing Gram-positive and *Klebsiella pneumoniae* representing Gram-negative organisms. Viable count of the test organisms was initially determined. A 0.5 ml volume of known cell density (by viable counts 10^6^ cfu/ml) from each organism suspension was added to 4.5 ml of different concentration of the fractions. The suspension was thoroughly mixed and held at room temperature (28–30°C) and the killing rate was determined over a period of 2 h. Exactly 0.5 ml of each suspension was withdrawn at the appropriate time intervals and transferred to 4.5 ml nutrient broth (Oxoid Ltd) recovery medium containing 3% “Tween 80” to neutralize the effects of the antimicrobial compounds carry-over from the test suspensions. The suspension was shaken properly then serially diluted up to 10^−5^ in sterile physiological saline. Exactly 0.5 ml of the final dilution of the test organism was transfer into pre-sterile nutrient agar (Oxoid Ltd) at 45°C and plated out. The plates were allowed to set and incubated upside down at 37°C for 72 h. Control experiment which was set up without the inclusion of antimicrobial agent. Viable counts were made in triplicates for each sample. Depression in the viable counts indicated killing by the antimicrobial agents.

### Determination of protein leakage from the test isolates by the active fraction

Cells of *Klebsiella pneumoniae* and *Enterococcus faecalis* from 18 h old nutrient broth culture were separately washed in 0.9% w/v NaCl (normal saline). Washed suspension of *Klebsiella pneumoniae* and *Enterococcus faec*alis (inoculums size approximately 10^6^ cells 0.5 McFarland standards) were treated with various concentration of the fractions relative to the MIC at various time interval for 2 h. Each suspension was then centrifuged at ≈ 5, 000 × g and supernatant collected was assayed for protein. Bradford [13] method of protein quantification was used to assay for protein: 0.4 ml Bradford reagent was added to 1.6 ml sample (0.2 ml supernatant plus 1.4 ml sterile distilled water) to make up to 2 ml total volume. Optical density (OD) of the resulting solution was thereafter taking at 595 nm after 5 min. The optical density of each of the samples was calculated from the equation of the best-fit linear regression line obtained from the graph of the Bovine Serum Albumin (BSA) standard curve.

### Determination of potassium ions leakage from the test isolates by the active fractions

The method of Allwood and Hugo [14] and Gale [15] was used for this assay. Cells of *Klebsiella pneumoniae* and *Enterococcus faecalis* from 18 h old nutrient broth culture were washed in 0.9% w/v NaCl (normal saline). Washed suspension of *Klebsiella pneumoniae* and *Enterococcus faecalis* (inoculums size approximately 10^6^ cells) were treated with various concentrations of the fractions relative to the MIC at various time interval for 2 h. Each suspension was then centrifuged at ≈ 11,000 × g and supernatant collected was assayed for potassium ions using atomic absorption spectroscopy. The amount of potassium ions leaked was calculated using standard curve (KCl standard curve). Sterile distilled water inoculated with the same quantity of inoculum was used as control.

### Determination of 260_nm_ absorbing materials leakage from the test isolates by the active fractions

The method described by Joswick [16] with modification was used to determine the leakage of the nucleotides from the test cells. Cells of *Klebsiella pneumoniae* and *Enterococcus faecalis* from 18 h old nutrient broth culture were washed in 0.9% w/v NaCl (normal saline). Washed suspension of *Klebsiella pneumoniae* and *Enterococcus faec*alis (inoculums size approximately 10^6^ cells) were treated with various concentration of the fractions relative to the MICs at various time intervals for 2 h. Each suspension was then centrifuged at ≈ 11,000 × g and the optical density of the supernatant collected was measured at 260_nm_ wave length using spectrophotometer. Sterile distilled water inoculated with the same quantity of inoculum was used as control.

### Statistical analyses

Data were expressed as means ± SD (standard deviation) of three replicates and were statistically analysed using one way analysis of variance (ANOVA). Means were separated by the Duncan multiple test using SAS. Values were considered significant at P < 0.05.

## Results

### The minimum inhibitory concentrations exhibited by extract, n-hexane, ethylacetate, n-butanol and aqueous fractions against susceptible bacterial isolates

The minimum inhibitory concentrations (MICs) of the extract and the active fractions obtained from the extract as well as that of streptomycin were shown in Table 1. The MIC of the extract exhibited against the susceptible bacterial isolates ranges between 0.39 mg/ml to 6.25 mg/ml. The MIC exhibited by the n-hexane, ethyl acetate and aqueous fractions ranges between 0.16 mg/ml and 5.00 mg/ml respectively while that obtained for n-butanol fraction was between 0.16 mg/ml and 2.50 mg/ml. The MIC of the standard antibiotic- streptomycin ranges between 0.30 mg/ml to 0.5 mg/ml.

**Table 1:** **The minimum inhibitory concentrations of the extract, n-hexane, ethylacetate, n-butanol and aqueous fractions and standard antibiotics exhibited against susceptible bacterial isolates**

**Bacterial isolates**	**CE (mg/ml)**	**N-HEX (mg/ml)**	**ETHYL (mg/ml)**	**BUT (mg/ml)**	**AQU (mg/ml)**	**STREP (mg/ml)**
*B. cereus* (NCIB 6349)	6.25	5.00	5.00	1.25	5.00	0.06
*B. polymyxa* (LIO)	6.25	ND	ND	ND	ND	0.13
*B. anthracis* (LIO)	6.25	2.50	5.00	2.50	5.00	0.25
*B. subtilis* (NCIB 3610)	6.25	5.00	5.00	2.50	5.00	0.25
*B. stearothermophillus* (NCIB 8222)	6.25	2.50	2.50	1.25	2.50	0.06
*C. sporogenes* (NCIB 532)	6.25	5.00	5.00	1.25	5.00	0.06
*C. pyogenes* (LIO)	6.25	5.00	5.00	2.50	5.00	0.13
*E. faecalis* (NCIB 775)	0.39	0.16	0.16	0.16	0.16	0.03
*M. luteus* (NCIB 196)	6.25	5.00	5.00	1.25	5.00	0.25
*K. pneumoniae* (NCIB 418)	6.25	5.00	5.00	1.25	5.00	0.50
*Ps. flourescens* (NCIB 3756)	3.13	5.00	5.00	1.25	5.00	0.03
*P. vulgaris* (LIO)	12.50	2.50	5.00	2.50	2.50	0.50

KEY: LIO = Locally Isolated Organism, NCIB = National Collection of Industrial Bacteria, CE = Plant extract, N-HEX = N-Hexane fraction, ETHYL = Ethyl acetate fraction, BUT = n-Butanol fraction, AQU = Aqueous fraction, STREP = Streptomycin, ND = Not Determined.

### The extent and rate of killing of *Klebsiella pneumoniae* and *Enterococcus faecalis* exhibited by n-hexane, ethylacetate, n-butanol and aqueous fractions

**Figure 1:**
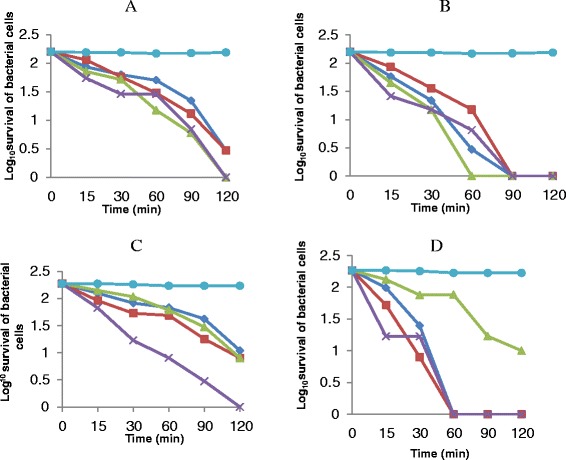
The extent and the rate of killing of *K. pneumoniae* **(A,** **B)** and *E. faecalis* **(C,** **D)** by n-hexane fraction (∆), ethylacetate fraction (×), n-butanol fraction (◊), aqueous fraction (□) and control **(**●**)** at 1 × MIC **(A,** **C)** and 2 × MIC **(B,** **D)**. Each point represents the log_10_ of mean survival of bacterial cells at a particular time interval in the presence of the fractions.

Two organisms were chosen for the killing rate test against the active fractions. *Klebsiella pneumoniae* was chosen to represent Gram negative, while *Enterococcus faecalis* represents Gram positive. The percentage of *K. pneumoniae* cells killed by n-hexane, ethylacetate, n-butanol and aqueous fractions at 1 × MIC after 15 min of contact time interval with each of the fraction were 54.4%, 65.2%, 45.6% and 27.8% respectively (Figure 1). By the end of 120 min, 100% of the cells were killed by n-hexane and ethylacetate fractions, while the percentage killed by n-butanol and aqueous fractions were 98.1% and 98.1% respectively. On the other hand, the percentages of *E. faecalis* cells killed by n-hexane, ethylacetate, n-butanol and aqueous fractions at the same concentration after 15 min of contact time interval were 24.2%, 64.0%, 33.3% and 51.1% respectively. At the end of 120 min contact time, the percentage of *E. faecalis* cells killed by n-hexane and aqueous was 95.0% each while that of ethyl acetate and n-butanol reached 100% at the same time interval. The killing rate reaction observed for n-hexane fraction against *E. faecalis* when the concentration to 2 × MIC follows the same trend as observed in 1 × MIC.

### The effect of the n-hexane, ethyl acetate, n-butanol and aqueous fractions on protein leakage from *Enterococcus faecalis* and *Klebsiella pneumoniae* cells

**Figure 2:**
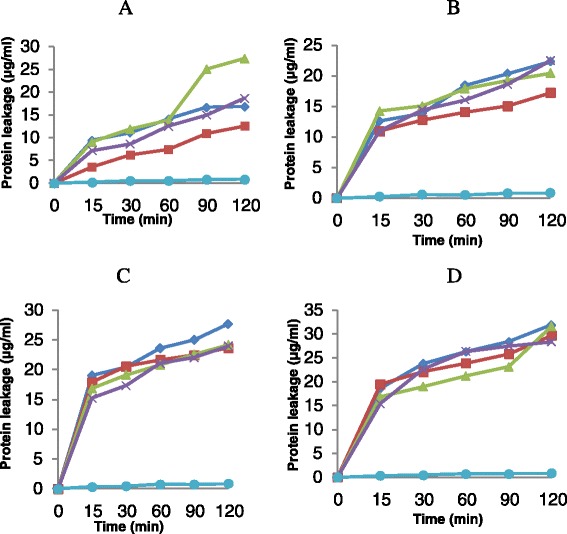
The effect of n-hexane fraction (◊), ethyl acetate fraction (□), n-butanol fraction (∆), aqueous fraction (×) and control (●) on protein leakage from *Enterococcus faecalis* **(A,** **B)** and *Klebsiella pneumoniae* **(C,** **D)** cells at 1 × MIC **(A, C)** and 2 × MIC **(B, D)**. Each point represents the amount of protein leaked (μg/ml) from the cells at a particular time interval in the presence of the fractions.

The amount of protein leaked by n-hexane fraction from *K. pneumoniae* cells after 120 min contact time were 27.71 μg/ml at 1 × MIC and 31.90 μg/ml at 2 × MIC. On the other hand, the amount of protein leaked from *E. faecalis* cells by n-hexane fraction at the end of 120 min contact time were 16.86 μg/ml at 1 × MIC and 22.23 μg/ml at 2 × MIC (Figure 2). The amount of protein leaked from *K. pneumoniae* cells by ethyl acetate, n-butanol and aqueous fractions followed the same trend as exhibited by n-hexane fraction against *E. faecalis.*

### The effect of n-hexane, ethyl acetate, n-butanol and aqueous fractions on potassium ion leakage from *Enterococcus faecalis* and *Klebsiella pneumoniae*

**Figure 3:**
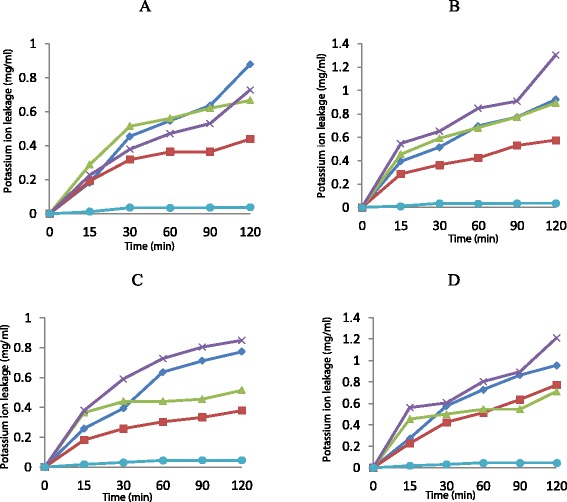
The effect of n-hexane fraction (◊), ethyl acetate fraction (□), n-butanol fraction (∆), aqueous fraction (×) and control (●) on potassium ions leakage from *Enterococcus faecalis* **(A,** **B)** and *Klebsiella pneumoniae* **(C,** **D)** cells at 1 × MIC **(A,** **C)** and 2 × MIC **(B, D)**. Each point represents the amount of potassium ions leaked (mg/ml) from cells at a particular time interval in the presence of the fractions.

From Figure 3, the concentration of the potassium ions leaked from *K. pneumoniae* cells at 1 × MIC by ethyl acetate fraction over a period of 15, 30, 60, 90 and 120 minutes were 0.182 mg/ml, 0.258 mg/ml, 0.303 mg/ml, 0.333 mg/ml and 0.379 mg/ml respectively. There were increases in the amounts of potassium ions leaked from the test cells when the concentration of the fractions were increased to 2 × MIC. The amount of potassium ions leaked from the test cells at this concentration over a period of 15, 30, 60, 90 and 120 minutes were 0.227 mg/ml, 0.424 mg/ml, 0.515 mg/ml, 0.636 mg/ml and 0.773 mg/ml respectively. Similar trends were observed when the isolates were tested against n-hexane, n-butanol and aqueous fractions at the same contact time intervals.

### The effect of n-hexane, ethyl acetate, n-butanol and aqueous fractions on 260_nm_ absorbing materials leakage from *Enterococcus faecalis* and *Klebsiella pneumoniae*

**Figure 4:**
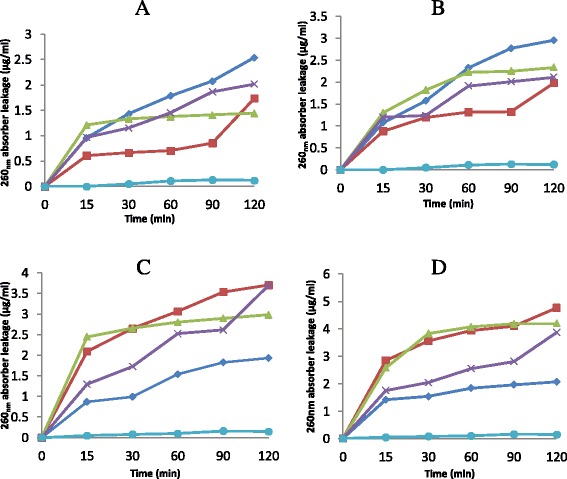
The effect of n-hexane fraction (◊), ethyl acetate fraction (□), n-butanol fraction (∆), aqueous fraction (×) and control (●) on 260 nm absorbing materials leakage from *Enterococcus faecalis* **(A,B)** and *Klebsiella pneumoniae* **(C,D)** at 1 × MIC **(A, C)** and 2 × MIC **(B, D)**. Each point represents the amount of nucleotides leaked (μg/ml) from the cells at a particular time interval in the presence of the fractions.

The leakage of the nucleotides from *K. pneumoniae* cells by n-hexane fraction after 15, 30, 60, 90 and 120 min at 1 × MIC were 0.870 μg/ml, 0.990 μg/ml, 1.539 μg/ml, 1.824 μg/ml and 1.931 μg/ml respectively. When the concentration of the fraction was increased to 2 x MIC the leakage observed after 15, 30, 60, 90 and 120 minutes were 1.415 μg/ml, 1.536 μg/ml, 1.838 μg/ml, 1.946 μg/ml and 2.070 μg/ml respectively (Figure 4).

## Discussion

The cytoplasmic membrane disruptions of *Enterococcus faecalis* and *Pseudomonas aeruginosa* were investigated in this study. The cytoplasmic membranes of bacteria provide barrier to the passage of materials such as antibiotics into the protoplasm. This permeability barrier role of cell membranes assist in solute transport, regulation of metabolism and control of turgor pressure [17]. Our findings revealed that minimum inhibitory concentrations exhibited by fractions obtained from *Cocos nucifera* husk extract led to the leakage of protoplasmic inclusion which include potassium ion, protein and nucleotides. Various MIC exhibited by these fractions are as indicated in Table 1 with the lowest MIC of 0.16 mg/ml exhibited by n-Butanol fraction against *Enterococcus faecalis*. Antimicrobial compound exhibiting MIC less than 100 mg/ml according to Holezt [18] has a good antimicrobial activity. This is an indication that n-Butanol fraction of *C. nucifera* extract exhibiting MIC below 100 mg/ml will be a veritable source of bioactive compounds. The kill rate assay of the fractions was carried out against test bacterial isolates as shown in Figure 1. Our observations revealed that the rate at which these fractions eliminate the cells increases with the concentration and contact time intervals. This supported results obtained by Rhodes [19] on antilisterial properties of red grape juice and red wine. The ability of an antibacterial agent to inhibit and kill bacteria is a measure of its effectiveness [20]. Results obtained from this study on the activities of the fractions against the test organisms by ethyl acetate fraction showed the highest activity and this is followed by n-hexane, butanol and aqueous fractions respectively.

Results obtained from this study indicate that these fractions leaked out appreciable amount of proteins from the test isolates as shown in Figure 2. In all cases, proteins leaked from these cells are directly proportional to the concentrations of the fraction and the time of exposure of these cells to the suspension of the fraction. The results obtained from this test was monophasic and similar to what was observed by Akinpelu and co-workers [21] in their studies on the effect of plant extract on microbial cell membrane. Zablotowics et al., [22] reported that saponins have the ability to cause leakage of proteins from bacterial cells. *Cocos nucifera* extract revealed the presence of this compound and thus might have also contributed to the leakage of protoplasmic materials observed in this study in addition to some other factors.

Leakages of potassium ions from the test isolates used for this study was a measure of probable way by which the fractions used for this study inhibit or kill the susceptible test isolates. These fractions exhibited reasonable potential to cause potassium efflux from the organisms (Figure 3). The concentration of potassium ion leaked from the test cells increases with an increase in the concentration of the fraction along with the contact time interval of the cells with the suspension of the fraction. The results obtained in this study is similar to the observation of AL-Adham et al. [23] in their studies on the effect of pyrithione biocides on the cell membrane disruption leading to potassium ion leakage from the cell.

Many antimicrobial compounds that act on the bacterial cytoplasmic membrane induce the loss of 260_nm_ absorbing materials [24]. The loss of these materials were observed in *Enterococcus faecalis* and *Pseudomonas aeruginosa* used for this study when treated with various fractions obtained from *C. nucifera* husk extract (Figure 3). Increase in concentrations of these fractions along with increase in contact time led to the loss of these nucleotides from the test cells. This is an indication of damage to the cell membrane of these organisms. Leakage of such materials from microbial cells when subjected to the effect of *Melaleuca alternifolia* oil was reported by Carson and co-workers [24].

There appears to be a relationship between the amount of cellular constituents released and the number of organisms killed as the contact time increased. For instance the percentage of *K. pneumoniae* cells killed by ethyl acetate fraction at 1 × MIC was 81.6% within 30 min of contact time while the amount of proteins leaked under the same condition was 20.59 μg/ml. When the contact time was increased to 120 min, 100% of the cells were killed and the amount of proteins leaked at the same time was 23.68 μg/ml. Thus, contact time and the amount of cellular materials leaked from the cell determined the population of the cells killed. In addition, the higher the concentrations of the fraction used the higher the number of cells killed. The results obtained from this study revealed that the extract from *Cocos nucifera* caused considerable amount of protoplasmic leakage and thus suggests that the extract have ability to cause damage to the cell membrane of the test organisms.

## Conclusions

The ability of *C. nucifera* extract to kill or inhibit Gram-positive and Gram-negative bacteria at low concentration and minimal contact time established the effectiveness of this extract as a potential source of broad spectrum antimicrobial drug. Such drug of natural origin may be used in the treatment of multidrug resistant pathogens. *Cocos nucifera* is used among the Yoruba tribe of Southwestern Nigeria for the preparation of decoction for consumption in folklore remedy and thus show the plant not to be toxic for human consumption. Therefore, different formulations could be prepared from this plant for clinical trials and such formulation could be more effective for the treatment of microbial infections.
